# Role of CXCR4 PET Imaging for the Evaluation of Gliomas: A Comprehensive Literature Review

**DOI:** 10.3390/cancers18162657

**Published:** 2026-08-17

**Authors:** Francesco Dondi, Pietro Bellini, Michela Cossandi, Roberto Rinaldi, Luca Camoni, Francesca Tomasoni, Massimo Statuto, Gian Luca Viganò, Francesco Bertagna

**Affiliations:** 1 Nuclear Medicine, ASST Spedali Civili di Brescia and University of Brescia, 25123 Brescia, Italy; francesco.bertagna@unibs.it; 2 Nuclear Medicine, ASST Spedali Civili di Brescia, 25123 Brescia, Italy; pietro.bellini@asst-spedalicivili.it (P.B.); michela.cossandi@asst-spedalicivili.it (M.C.); roberto.rinaldi@asst-spedalicivili.it (R.R.); francesca.tomasoni@asst-spedalicivili.it (F.T.); massimo.statuto@asst-spedalicivili.it (M.S.); 3 Allied Health and Social Care Professions Directorate, ASST Spedali Civili di Brescia, 25123 Brescia, Italy; luca.camoni@unibs.it; 4 Clinical Engineering, ASST Spedali Civili di Brescia, 25123 Brescia, Italy; gianluca.vigano@asst-spedalicivili.it

**Keywords:** CXCR4, PET, positron emission tomography, PET/CT, glioma, glioblastoma, [68Ga]Ga-pentixafor, brain tumors, neuro-oncology, NOTA

## Abstract

C-X-C chemokine receptor type 4 (CXCR4)-targeted positron emission tomography (PET) radiotracers have recently emerged as promising molecular imaging tools for glioma evaluation because of their negligible physiological uptake in normal brain tissue and their ability to non-invasively assess CXCR4 expression. This narrative review summarizes the current evidence regarding their clinical applications in glioma imaging. a literature review was performed to identify clinical studies investigating CXCR4-targeted PET in glioma patients. The available evidence, although limited and heterogeneous, indicates that CXCR4-targeted PET tracers can provide high lesion-to-background contrast because of their minimal physiological uptake in normal brain tissue. Tracer uptake tends to be higher in high-grade gliomas, although correlations with histopathological CXCR4 expression and established prognostic biomarkers have been inconsistent. Preliminary studies have explored potential applications in tumor visualization, differentiation of recurrence from treatment-related changes, distinction between glioblastoma and primary central nervous system lymphoma, treatment response assessment, and radiotherapy planning. These findings require validation in larger prospective multicenter cohorts. CXCR4-targeted PET is an emerging molecular imaging approach that may provide complementary information in glioma assessment. However, the available evidence remains preliminary, and larger prospective multicenter studies are required to validate its diagnostic and clinical utility, standardize imaging and quantification protocols, and determine its role in routine neuro-oncological practice.

## Simple Summary

Gliomas are among the most aggressive brain tumors and remain difficult to diagnose, monitor, and treat effectively. Current imaging methods provide important information but may not fully capture the biological processes that drive tumor growth, treatment resistance, and recurrence. This review summarizes the available clinical evidence on positron emission tomography targeting the C-X-C chemokine receptor type 4, a protein involved in glioma progression and interactions with the tumor microenvironment. The findings suggest that CXCR4-targeted imaging can identify aggressive tumor areas because of its very low uptake in normal brain tissue and may complement conventional magnetic resonance imaging and established positron emission tomography tracers. Although current evidence is still limited, this approach may improve tumor characterization, help assess treatment response, support personalized treatment planning, and facilitate the development of future targeted therapies. Larger prospective studies are needed before routine clinical use.

## 1. Introduction

Gliomas are the most common malignant primary tumors of the central nervous system and are characterized by marked molecular and biological heterogeneity [1,2,3,4]. The current World Health Organization (WHO) classification highlights the importance of molecular alterations in defining tumor entities, prognosis, and therapeutic strategies [5]. Among diffuse gliomas, glioblastoma (GBM), particularly isocitrate dehydrogenase (IDH)-wildtype GBM, remains the most aggressive subtype, with poor survival outcomes despite advances in multimodal treatments including surgery, radiotherapy, chemotherapy, and targeted approaches. Tumor recurrence, diffuse infiltration, and therapeutic resistance continue to represent major challenges in clinical management [6,7,8,9,10,11,12,13,14,15].

Neuroimaging plays a central role in the diagnosis and follow-up of glioma patients. Magnetic resonance imaging (MRI) remains the standard imaging modality providing detailed anatomical information and advanced functional parameters, such as perfusion and diffusion metrics, which support the identification of aggressive forms, and which may also be able to differentiate tumor progression from treatment-related effects, including pseudoprogression and radiation necrosis [16,17,18,19,20,21].

Positron emission tomography (PET) with targeted radiotracers has emerged as a valuable tool for the assessment of a wide range of pathological conditions that may affect the nervous system [22,23,24]. In particular, in the specific setting of neuro-oncology, this imaging modality allows for the in vivo assessment of neoplasms and the visualization of specific biological processes that may be involved in tumor progression [25,26,27]. Amino acid PET is an established imaging modality in neuro-oncology and provides clinically relevant information on tumor extent, treatment response, and suspected recurrence. Nevertheless, tracer uptake may also occur in non-neoplastic inflammatory or treatment-related conditions, and amino acid tracers do not directly characterize specific oncogenic pathways or receptor expression [28,29,30,31]. Despite that, several other radiopharmaceuticals have been investigated for this purpose [17,32,33,34,35,36,37,38].

Among the molecular targets investigated, the C-X-C chemokine receptor type 4 (CXCR4) has gained increasing interest due to its involvement in progression and interaction with the disease microenvironment in different types of pathological conditions, including neoplasms [39,40,41,42,43,44,45]. CXCR4 is a G protein-coupled receptor activated by C-X-C motif chemokine ligand 12 (CXCL12), and this signaling axis is involved in physiological processes such as immune cell trafficking and stem cell migration. In cancer, dysregulation of CXCR4/CXCL12 signaling promotes tumor cell migration, invasion, angiogenesis, immune modulation, and resistance to therapy [39,46,47,48,49,50,51,52,53]. In gliomas, CXCR4 expression has been identified in tumor cells and microenvironmental components, including endothelial and immune cells. Its activation has been associated with increased invasiveness, maintenance of glioma stem-like cells, and a more aggressive tumor phenotype [54,55,56,57,58,59,60,61,62]. These features made therefore CXCR4 a promising biomarker for molecular imaging and a potential therapeutic target.

The development of CXCR4-targeted PET radiotracers has enabled non-invasive visualization of CXCR4 expression in vivo. Initially investigated in hematological malignancies, CXCR4-directed radiopharmaceuticals, including peptide-based ligands and small-molecule antagonists, are now being explored in solid tumors and primary brain tumors [63,64,65,66,67,68]. Preliminary studies suggest that CXCR4 PET may provide complementary biological information beyond conventional imaging and could support tumor characterization, patient selection for targeted therapies, and future theranostic approaches [69,70,71,72,73,74]. However, the clinical role of CXCR4-targeted PET in gliomas is still under investigation and remains exploratory. The aim of this narrative review is to summarize the available clinical evidence on CXCR4-targeted PET imaging in patients with gliomas, including its potential applications in tumor characterization, differential diagnosis, recurrence assessment, treatment monitoring, and radiotherapy planning.

## 2. Materials and Methods

A literature search was performed to identify clinical studies investigating the application of PET imaging with CXCR4-targeted radiopharmaceuticals in patients with gliomas. The search was carried out in three electronic databases—PubMed/MEDLINE, Scopus, and Embase—which were selected for their comprehensive coverage of biomedical and clinical research.

**Figure 1:**
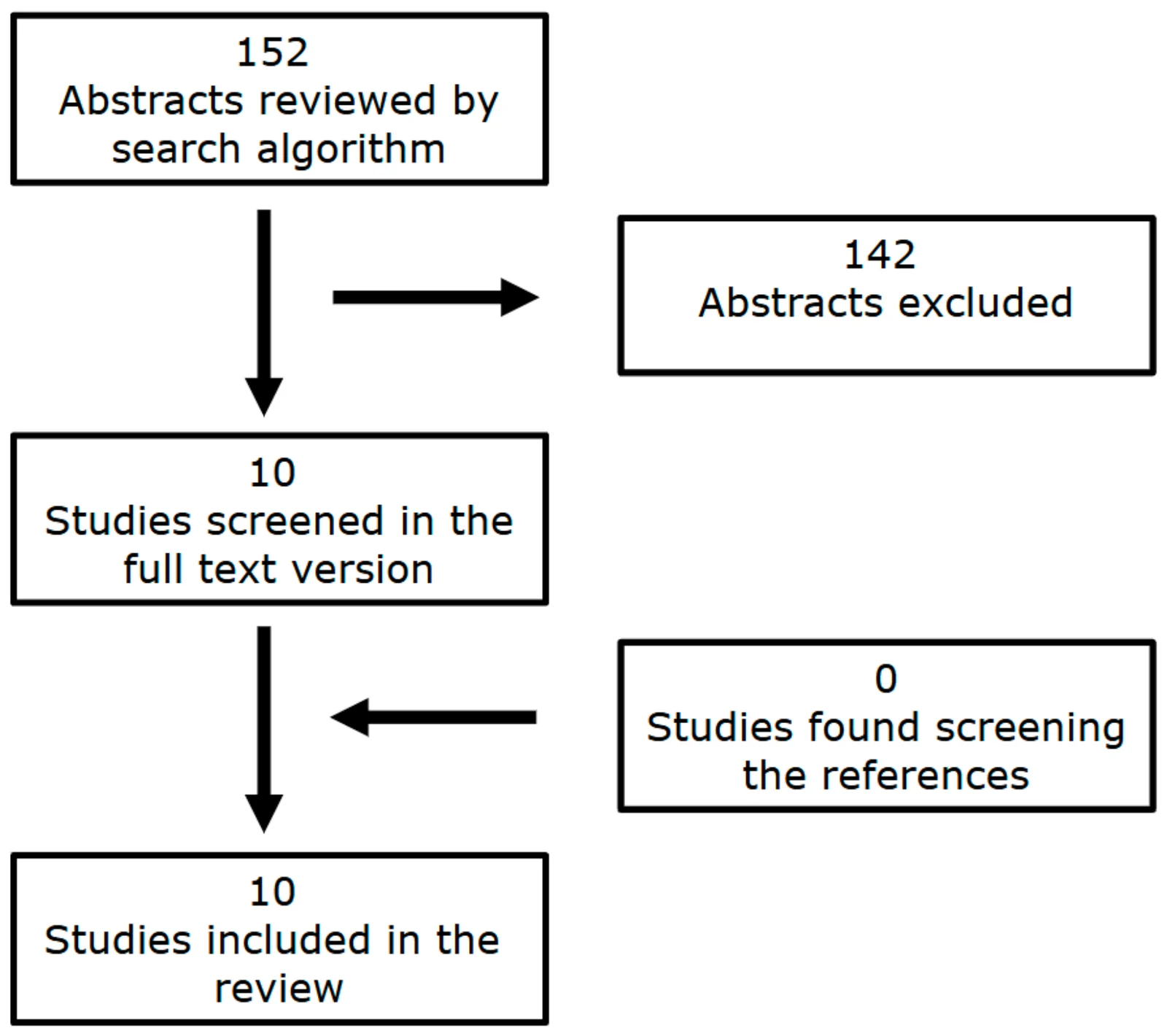
Flowchart of the research of eligible studies included in the review.

The search string used was (glioma OR glioblastoma OR GBM OR astrocytoma OR oligodendroglioma OR “diffuse glioma” OR “high-grade glioma” OR “low-grade glioma”) AND (CXCR4 OR “chemokine receptor 4” OR “C-X-C chemokine receptor 4” OR Pentixafor OR Pentixather OR “68Ga-Pentixafor” OR “Ga-68 Pentixafor” OR “68Ga-Pentixather” OR “177Lu-Pentixather” OR “64Cu-Pentixafor” OR “CXCR4”) AND (PET OR “PET/CT” OR “positron emission tomography”). The search included all eligible publications available from database inception through 1 July 2026, without restrictions on the year of publication. Only articles written in English were considered. To ensure that the review was based on primary clinical evidence, studies were excluded if they were preclinical or in vitro investigations, conference abstracts, review articles, editorials, letters, case reports or other publications lacking original clinical data. Consequently, only original clinical studies assessing the clinical role of CXCR4-targeted PET imaging in patients with glioma—including tumor detection and characterization, differential diagnosis, recurrence evaluation, treatment response assessment, and radiotherapy planning—were included in the final analysis. A total of 227 papers were extracted after the database search and, after removing duplicates, 152 articles were finally evaluated. After applying the aforementioned exclusion criteria, 10 papers were selected to be included in the present review (Figure 1).

The review was structured in accordance with the quality domains proposed by SANRA for narrative review articles. These domains include justification of the topic’s relevance, a clear definition of the review objectives, a transparent description of the literature search strategy, sound scientific reasoning, a critical appraisal of the available evidence, and comprehensive reporting of relevant outcome data [75]. Key methodological aspects were systematically extracted and reported to facilitate a critical evaluation of the available evidence. For each study, the imaging modality, scanner model, administered activity, uptake time, reconstruction protocol, definition of background activity, and tumor-to-background ratio (TBR) calculation method were extracted when available.

**Figure 2:**
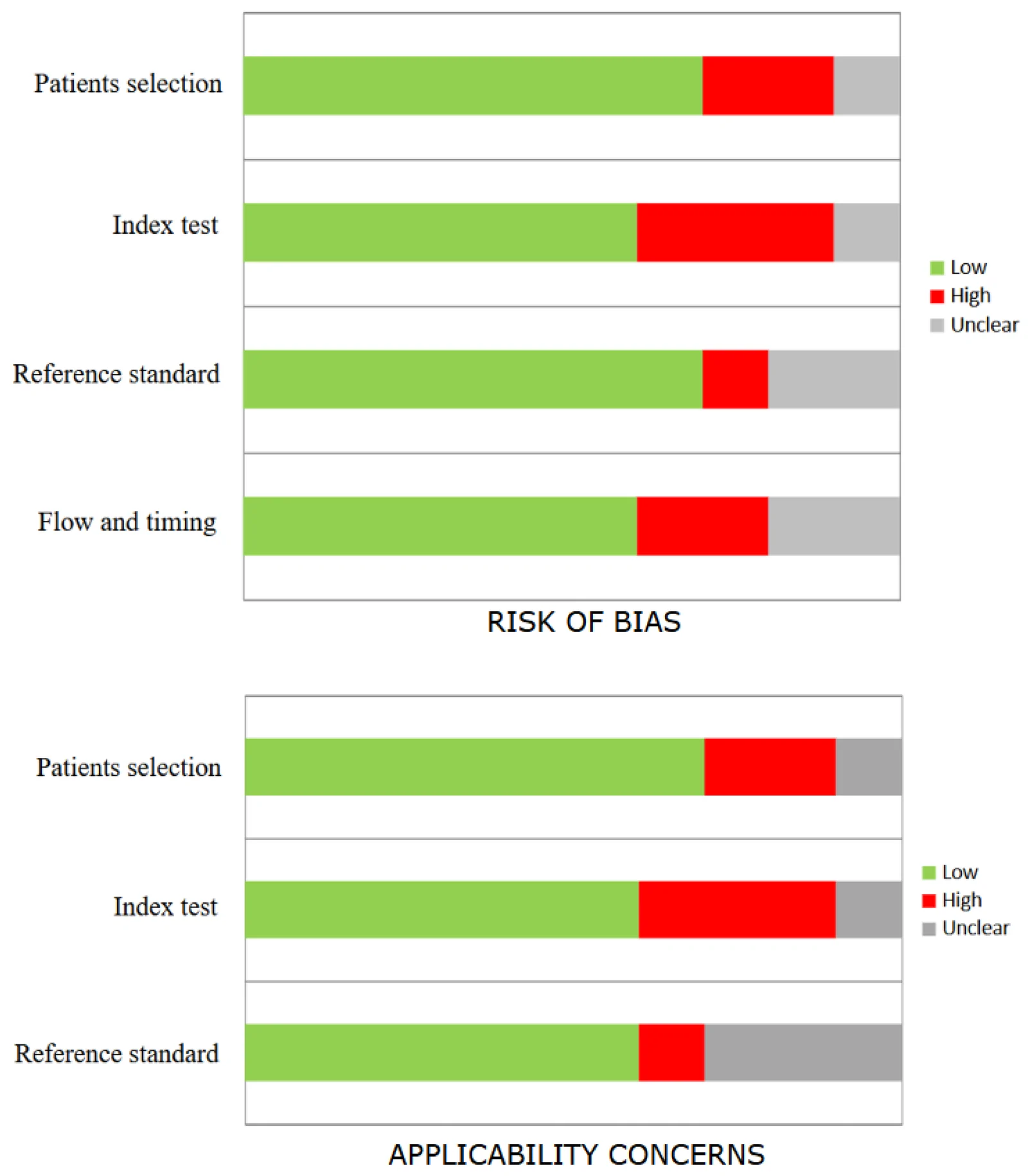
QUADAS-2 quality assessment for risk of bias and applicability concerns for the studies included in the review.

The quality assessment of these studies, including the risk of bias and applicability concerns, was carried out using the Quality Assessment of Diagnostic Accuracy Studies version 2 (QUADAS-2) evaluation [76]. Overall, the risk-of-bias assessment showed a generally favorable methodological profile across the included studies. The majority of studies were judged to have a low risk of bias across all QUADAS-2 domains, including patient selection, index test, reference standard, and flow and timing. Only a limited number of studies were considered to have a high risk of bias in individual domains. Therefore, although some methodological concerns were identified, these were relatively uncommon and did not affect the overall assessment of most studies, supporting the robustness of the available evidence (Figure 2).

## 3. The Role of CXCR4 Radiotracers for the Evaluation of Gliomas

Different studies assessed the added clinical value of CXCR4 radiotracers in the evaluation of glioma patients [77,78,79,80,81,82,83,84,85,86] (Table 1, Table 2, Table 3 and Table 4).

### 3.1. Gliomas Uptake

The first instance of CXCR4 PET imaging in gliomas was carried out using [68Ga]NOTA-NFB, which demonstrated negligible uptake in the normal brain, resulting in a very low absorbed radiation dose to brain tissue and indicating that the radiotracer did not cross an intact blood–brain barrier [77].

When focusing on GBM, [68Ga]Ga-pentixafor PET showed low to moderate uptake in the tumor (mean standardized uptake value (SUV)mean 1.45 and mean SUVmax 2.06) compared with blood-pool activity (mean SUVmean 1.36). Despite that, the very low background activity (mean SUVmean 0.03 and mean SUVmax 0.10) led to a relatively high TBR (mean TBRmean 67.0, mean TBRmax 65.6) even in the presence of low to moderate uptake of the primary neoplasm [79]. The same results were also confirmed in other reports, where all subjects showed clear tracer uptake in the primary tumors, with mean SUVmax, SUVmean and TBR of 4.5 ± 1.6, 0.60 ± 0.26 and 6.9 ± 4.6, respectively [83]. In this setting, the same radiotracer demonstrated high diagnostic performance when evaluating HGG, with a positive PET in 25 of 26 patients and in 27 of 28 lesions, corresponding to patient-based and lesion-based detection rates of 96.2% and 96.4%, respectively [82]. Focusing again on HGG, at both staging or after recurrence of disease, [68Ga]Ga-pentixafor imaging was positive in 27/29 subjects, with a median SUVmax of 2.31 and a median TBR of 20 [84]. Interestingly, it has been suggested that when performed together, MRI showed typical GBM features, and a significant correlation (*p* < 0.005) was observed between [68Ga]Ga-pentixafor SUVmean and the choline/N- acetylaspartate ratio [83].

### 3.2. Comparison with Other Radiotracers

Unlike [18F]FDG, which showed intense physiological uptake in normal brain tissue, [68Ga]NOTA-NFB exhibited almost no background activity in the healthy brain (SUVmax 0.11 ± 0.02), providing excellent TBR. Although the absolute gliomas SUVmax was lower than that of [18F]FDG (4.11 ± 2.90 vs. 7.34 ± 2.90 respectively, *p* < 0.05), the TBR was significantly higher (9.21 ± 8.75 vs. 0.86 ± 0.41 respectively, *p* < 0.05), allowing for a more accurate delineation of tumor margins [77]. Similarly, it has also been reported that, in a patient with low-grade glioma (LGG) imaged with both [18F]AlF-NOTA-QHY-04 and [18F]FDG, [18F]AlF-NOTA-QHY-04 showed a lower SUVmax but a higher TBR than [18F]FDG [80].

When using both [18F]fluoroethyl-l-tyrosine ([18F]FET) and [68Ga]Ga-pentixafor, in a cohort of 15 patients with suspected primary or recurrent high-grade glioma, [68Ga]Ga-pentixafor PET was positive in 13/15 lesions. Among the 13 patients ultimately diagnosed with high-grade glioma, [68Ga]Ga-pentixafor uptake was observed in 11 (11/13). The two additional [68Ga]Ga-pentixafor-positive lesions were a primary central nervous system lymphoma and a post-stroke inflammatory lesion. [18F]FET PET was available in 11/15 patients and was positive in all examined cases. Absolute uptake was higher with [18F]FET than with [68Ga]Ga-pentixafor (SUVmean: 4.4 ± 2.0 vs. 3.0 ± 1.5, *p* = 0.05; SUVmax: 5.3 ± 2.3 vs. 3.9 ± 2.0, *p* = 0.10). However, because of the lower physiological background activity, [68Ga]Ga-pentixafor yielded significantly higher TBRs (*p* < 0.01). The highest [68Ga]Ga-pentixafor uptake was observed predominantly in contrast-enhancing tumor regions, and the spatial distributions of [68Ga]Ga-pentixafor and [18F]FET differed in most cases [78].

When comparing [18F]NOTA-NFB PET/MRI in the clinical setting of glioma recurrence detection with [11C]methionine ([11C]MET) PET/MRI and contrast-enhanced MRI (ceMRI), the accuracy of MRI (76.32%) was significantly lower than that of [18F]NOTA-NFB PET imaging (92.11%, *p* 0.031) and [11C]MET PET imaging (94.74%, *p* = 0.039) in distinguishing recurrent gliomas from treatment-related effects, although the difference was not statistically significant between the two radiotracers (*p* = 1.0). The same results were confirmed also when focusing on specificity, while the sensitivity for all modalities was 100%. Both [18F]NOTA-NFB and [11C]MET PET showed significantly higher SUVmax (2.20 vs. 0.79 and 3.65 vs. 1.77, *p* < 0.01 in both cases) and TBR (43.33 vs. 16.67 and 3.34 vs. 1.50, *p* < 0.01 in both cases) in recurrent tumors, compared with treatment-related effects. Although the uptake of both tracers increased in recurrent tumors, [18F]NOTA-NFB imaging demonstrated significantly lower SUVmax compared with [11C]MET PET (2.20 vs. 3.65, *p* < 0.001). Moreover, [18F]NOTA-NFB showed a significantly higher TBR for recurrence than [11C]MET (43.33 vs. 3.34, *p* < 0.001). [11C]MET SUVmax achieved an AUC of 0.911, with sensitivity, specificity, positive predictive value (PPV), negative predictive value (NPV) and accuracy of 0.90, 0.93, 0.82, 0.96, and 0.92, respectively. [18F]NOTA-NFB SUVmax showed an AUC of 0.793, with sensitivity, specificity, PPV, NPV, and accuracy of 0.80, 0.89, 0.73,0.93, and 0.87, respectively. [11C]MET TBR had an AUC of 0.889, while [18F]NOTA-NFB TBR had an AUC of 0.829. No significant differences were reported between the diagnostic performances of such parameters [86].

### 3.3. Glioma Grading

In general, it has been reported that HGG had higher CXCR4 traces uptake compared with LGG [77]. In particular, significantly higher tracer uptake in high-grade tumors was reported: WHO grade IV lesions had a median SUVmax of 3.03, compared to 1.51 in grade III tumors (*p* = 0.0145). Similarly, TBRs were significantly higher in grade IV gliomas (mean value 2.1) compared to grade III lesions (mean value 0.75, *p* = 0.0002). Visual and semi-quantitative assessments showed higher uptake in tumor tissue compared to contralateral brain parenchyma and blood pool, particularly in grade IV neoplasms, where uptake ranged from moderate to intense [82]. Similar findings were also reported in other papers, where median SUVmax of grade IV lesions was significantly higher than that of grade III lesions (2.68 vs. 1.51, *p* = 0.02); however, there was no significant difference between grade IV and grade III TBR (20.0 vs. 22.37) [84]. In this setting, it has been reported that CXCR4 expression increased with glioma grade, with the highest but highly heterogeneous levels observed in GBM, while normal brain tissue was consistently CXCR4-negative [79,83].

### 3.4. Histopathology

Histopathological analysis generally confirmed the presence of CXCR4 expression in regions with tracer uptake. In resected tumors stronger immunohistochemical staining was observed in HGG, which corroborated the SUVmax of [68Ga]NOTA-NFB PET imaging [77]. Similar findings were reported for [68Ga]Ga-pentixafor, where tumors with weak CXCR4 expression were PET-negative, whereas areas with high [68Ga]Ga-pentixafor uptake corresponded to regions with moderate or strong CXCR4 expression [78]. Despite that, in another report, even though all patients showed large intratumor variation for CXCR4 staining, it did not fully correspond with variation in tracer uptake [79]. Yet among tumors with moderate to high CXCR4 expression, the intensity of tracer uptake did not correlate quantitatively with immunohistochemical CXCR4 scores (*p* = 0.316). Similarly, no significant association was observed between tracer uptake, CXCR4 expression and Ki-67 expression [78]. In this setting, it has also been reported that neither CXCR4 mRNA nor protein expression correlated with IDH status, O6-Methylguanine-DNA methyltransferase (MGMT) promoter methylation, or overall survival, limiting its value as an independent prognostic biomarker [79].

In an interesting paper that compared the [68Ga]Ga-pentixafor uptake in different neoplasms, specimens analysis showed high CXCR4 positivity in both primary central nervous system lymphoma (PCNSL) and GBM (91.4% for PCNSL vs. 90.9% for GBM); however, its expression was significantly higher in PCNSL (*p* = 0.048). SUVmax correlated strongly with CXCR4 staining in PCNSL (r 0.717, *p* < 0.001) and more weakly in GBM (r 0.543, *p* = 0.002) [85].

### 3.5. Differential Diagnosis with Other Neoplasms

Cheng et al. [80] compared [18F]AlF-NOTA-QHY-04 and [18F]FDG for the evaluation of different hematological and solid neoplasms, also including 7 patients with gliomas. Different patterns of disease extent and uptake were demonstrated across different types of neoplasms; the [18F]AlF-NOTA-QHY-04 uptake of non-small cell lung cancer, triple negative breast cancer, pancreatic cancer, glioma, hepatocellular carcinoma and colorectal cancer were all significantly lower than small cell lung cancer (SCLC) (*p* < 0.05), and showed no significant differences between each other (*p* > 0.05). Focusing on brain lesions, the average uptake of primary central nervous system diffuse large B-cell lymphoma was the highest, followed by HGG (7.39 ± 1.65 vs. 3.13 ± 0.58 respectively, *p* < 0.01). There were no significant differences between LGG and brain metastases of SCLC (1.18 ± 0.51 vs. 1.27 ± 0.18 respectively, *p* = 0.716), both significantly lower than HGG (*p* < 0.01). Interestingly, the results of [18F]AlF-NOTA-QHY-04 were useful in patients who underwent repeated biopsy due to imaging suggestive of HGG.

A similar paper by Chen et al. [85] assessed the value of [68Ga]Ga-pentixafor PET imaging in distinguishing PCNSL from GBM. Most GBM patients (51/53) had PET-positive lesions, and the rate for positivity was not significantly different between PCNSL and GBM (*p* = 0.181). PCNSL and atypical PCNSL had significantly higher SUVmax and TBR and lower metabolic tumor volume (MTV) values compared to GBM (*p* < 0.05 in all cases). A threshold of 67.1 of TBR had the highest diagnostic performance with an area under the curve (AUC) of 0.804 (*p* < 0.001) in differentiating between PCNSL and GBM (sensitivity 71.8% and specificity 83.0%). SUVmax, MTV, apparent diffusion coefficient (ADCmin) and relative ADCmin had AUCs of 0.774, 0.690, 0.800 and 0.726, respectively. In differentiating between atypical PCNSL and GBM, TBR confirmed its highest performance (AUC of 0.784, 82.6% sensitivity and 75.5% specificity). In differentiating PCNSL from GBM, TBR (odds ratio (OR) 1.031, *p* < 0.001), MTV (OR 0.937, *p* = 0.023) and ADCmin (OR 0.989, *p* < 0.001) were reported as independent predictors. The combined quantitative model achieved an AUC of 0.913 (sensitivity 84.5%, specificity 86.8%), significantly outperforming single parameters (*p* < 0.010). When adding qualitative MRI features, significant independent predictors included TBR (OR 1.035, *p* < 0.001), MTV (OR 0.933, *p* = 0.024), ADCmin (OR 0.988, *p* < 0.001), number of lesions (OR 5.947, *p* = 0.007), and necrosis (OR 0.109, *p* = 0.001). The resulting multiparameter model reached an AUC of 0.953 (sensitivity 87.3%, specificity 88.7%, *p* < 0.001). When differentiating atypical PCNSL from GBM, TBR (OR 1.026, *p* = 0.002) and ADCmin (OR 0.989, *p* = 0.002) were reported as independent factors. The combined quantitative model showed an AUC of 0.883 (sensitivity 91.3%, specificity 73.6%). In an extended model including lesion number and enhancement patterns, TBR (OR 1.029, *p* = 0.001), ADCmin (OR 0.989, *p* = 0.003), and lesion number (OR 5.845, *p* = 0.017) remained significant predictors, yielding an AUC of 0.902 (sensitivity 82.6%, specificity 86.7%; *p* < 0.001).

### 3.6. Treatment Guidance and Planning

[68Ga]Ga-pentixafor PET/CT was used to assess patients before and after chemo-radiation (C-RT) and, at baseline, mean SUVmax, SUVmean and TBR were 4.4 ± 1.6, 0.65 ± 0.32 and 5.96 ± 3.5, respectively [83]. After the completion of therapy, the values were similar for SUVmax and SUVmean (4.6 ± 2.1 and 0.60 ± 0.31 respectively), while TBR increased significantly to 8.84 ± 5.72 (*p* = 0.05), suggesting improved lesion contrast or evolving tumor biology. In this setting, in the group of patients with stable disease, PET parameters tended to decrease or remain stable, with baseline vs. follow-up values of SUVmax 3.70 ± 0.90 vs. 2.64 ± 1.34, SUVmean 0.40 ± 0.12 vs. 0.31 ± 0.24 and TBR 4.4 ± 2.28 vs. 2.91 ± 0.93. In subjects with progressive disease, SUVmax increased from 4.70 ± 1.82 to 5.62 ± 1.65 and TBR increased from 6.75 ± 3.80 to 8.75 ± 3.68, whereas SUVmean changed from 0.77 ± 0.32 to 0.73 ± 0.26. Therefore, longitudinal changes were not uniform across all PET-derived parameters [83].

Lastly, Madan et al. [81] published an interesting paper aimed to assess whether [68Ga]Ga-pentixafor PET imaging could be useful to guide radiotherapy dose escalation in grade IV gliomas in a prospective phase II study. PET-guided dose escalation was feasible, and neurological adverse events were predominantly grade I or II; one patient developed grade III neurological toxicity. However, no survival benefit was demonstrated.

## 4. Discussion

The available evidence indicates that CXCR4-targeted PET imaging represents a promising molecular imaging approach for the evaluation of gliomas, providing information that is complementary to conventional anatomical imaging and to currently established PET radiotracers [87,88,89,90,91,92,93]. Although the number of published studies remains limited and most investigations are based on relatively small, single-center cohorts, the overall findings seem to demonstrate that CXCR4-directed tracers can visualize biologically aggressive tumor components with excellent lesion conspicuity due to their negligible physiological uptake in normal brain tissue [77,78,79,80,81,82,83,84,85,86]. In this setting, one of the most consistent observations across studies is the remarkably low background activity of CXCR4 radiotracers within the healthy brain. Both [68Ga]Ga-pentixafor and the newer NOTA-based compounds ([68Ga]NOTA-NFB, [18F]AlF-NOTA-QHY-04 and [18F]NOTA-NFB) showed minimal physiological uptake in normal cerebral tissue [77,78,79,82,83,86]. Consequently, despite generally lower absolute SUV values compared to other mentioned tracers, CXCR4-targeted PET consistently achieved substantially higher TBRs [77,78,80,86]. This feature may facilitate the visualization of selected tumor regions and improve TBR contrast. However, current evidence does not establish that CXCR4-targeted PET can reliably delineate non-enhancing infiltrative glioma.

Another recurrent finding is the association between CXCR4 tracer uptake and glioma grade. Most studies reported significantly higher uptake in WHO grade IV gliomas compared with grade III tumors, whereas LGG generally demonstrated lower tracer accumulation [77,79,80,82,84]. Histopathological analyses further confirmed that CXCR4 expression increases with tumor aggressiveness, supporting the biological rationale for CXCR4 imaging as a marker of malignant transformation [77,79,83,85]. These observations are consistent with the established role of the CXCL12/CXCR4 signaling axis in glioma progression. Indeed, increasing evidence indicates that this pathway plays a central role in glioma biology by promoting tumor growth, angiogenesis, cell invasion, maintenance of glioma stem-like cells, and resistance to conventional therapies, while also contributing to the dynamic interactions between tumor cells and the surrounding microenvironment [94,95,96,97,98]. Nevertheless, substantial intratumoral heterogeneity of CXCR4 expression was observed in some studies [78,79,84]. This heterogeneous distribution likely reflects the complex biological organization of GBM, where different tumor regions exhibit variable degrees of hypoxia, necrosis, vascular proliferation, and inflammatory infiltration [99,100,101,102]. Such heterogeneity may explain why PET uptake frequently identifies only selected portions of the contrast-enhancing lesion rather than the entire tumor volume. Because the highest [68Ga]Ga-pentixafor uptake was observed predominantly in contrast-enhancing regions, its ability to identify infiltrative tumor beyond the contrast-enhancing volume remains to be demonstrated.

Although immunohistochemistry generally confirmed CXCR4 expression in PET-positive lesions, the quantitative relationship between imaging findings and tissue expression remains inconsistent and evidence from the studies is heterogenous. Wang et al. demonstrated a clear association between increasing tracer uptake and stronger CXCR4 staining [77], whereas Lapa et al. found that PET-negative tumors exhibited weak receptor expression but failed to demonstrate a quantitative correlation between PET uptake and immunohistochemical scores among CXCR4-positive tumors [78]. Similarly, Jacobs et al. reported marked intratumoral heterogeneity without complete correspondence between tracer uptake and immunohistochemistry [79], while Chen et al. observed a significant correlation between SUVmax and CXCR4 expression that was stronger in PCNSL than in GBM [85]. Furthermore, no consistent associations were identified between PET parameters and other immunohistochemistry parameters such as Ki-67, IDH mutation status or MGMT promoter methylation [78,79]. Collectively, these findings suggest that CXCR4 PET primarily reflects the spatial distribution of receptor expression rather than serving as a direct quantitative surrogate of receptor density or tumor proliferation. Methodological factors, including sampling bias due to intratumoral heterogeneity and differences between biopsy location and PET-derived tumor volumes, may also contribute to these discrepancies. Importantly, tracer uptake should not be interpreted as a direct measure of CXCR4 receptor density alone. Signal intensity may also be influenced by blood–brain barrier integrity, tumor perfusion and tracer delivery, vascular or blood-pool activity, partial-volume effects, and spatial mismatch between PET-derived volumes and histological samples. Moreover, the uptake reported in PCNSL and in non-neoplastic inflammatory tissue indicates that CXCR4-targeted PET is not specific for glioma.

Comparison with other PET radiotracers provides further insight into the potential role of CXCR4 imaging. In this setting, several radiopharmaceuticals are currently used for glioma imaging, including amino acid-based tracers such as [18F]FET, [11C]MET, and [18F]FDOPA, which allow for the visualization of tumor extent and biological activity [103,104,105,106,107] (Table 5).

As previously mentioned, relative to [18F]FDG, CXCR4-targeted tracers offer a major advantage because of the absence of physiological cortical uptake, resulting in markedly improved lesion contrast despite lower absolute tracer accumulation [77,80]. Compared with amino acid tracers such as [18F]FET and [11C]methionine, however, the picture is more nuanced. Amino acid PET generally demonstrated higher absolute uptake and slightly higher sensitivity for tumor detection, whereas CXCR4 PET consistently achieved higher TBR owing to its extremely low physiological brain uptake [78,86]. Importantly, the limited spatial overlap observed between [68Ga]Ga-pentixafor and [18F]FET uptake suggests that these tracers interrogate distinct biological processes rather than providing redundant information [78]. While amino acid PET primarily reflects increased amino acid transport and protein synthesis, CXCR4 imaging may identify receptor overexpression associated with invasive behavior, hypoxia, stem cell niches, and tumor–microenvironment interactions [60,108]. Consequently, these techniques should be regarded as complementary rather than competitive imaging modalities.

As mentioned, CXCR4 PET imaging has been used to assess the presence or to possibly endorse the diagnosis of gliomas. Additionally, several studies explored different clinical applications of CXCR4 imaging. In recurrent gliomas, [18F]NOTA-NFB PET demonstrated significantly higher diagnostic accuracy than ceMRI for distinguishing tumor recurrence from treatment-related changes and achieved diagnostic performance comparable to [11C]MET imaging [86]. As mentioned, the high TBR of [18F]NOTA-NFB facilitated lesion detection despite relatively modest SUV values, highlighting its potential utility in one of the most challenging scenarios in neuro-oncology [107,108,109,110,111]. Likewise, preliminary evidence suggests that serial [68Ga]Ga-pentixafor PET may contribute to treatment response assessment following C-RT, as patients with stable disease generally showed stable or decreasing PET-derived parameters, whereas patients with progressive disease showed increases in SUVmax and TBR, but not in SUVmean [83]. Another promising application, despite being explored in only a single paper, concerns radiotherapy planning, where the heterogeneous intratumoral distribution of CXCR4 expression raises the possibility of identifying biologically aggressive tumor subvolumes suitable for dose escalation [81]. However, despite the feasibility of biologically guided dose escalation, no improvement in survival outcomes was demonstrated, indicating that further investigation in larger prospective studies is warranted.

The theranostic implications of CXCR4 imaging also deserve consideration. Because [68Ga]Ga-pentixafor has therapeutic counterparts labeled with β-emitting radionuclides, non-invasive assessment of receptor expression could potentially identify patients eligible for CXCR4-targeted radionuclide therapy. Although this strategy has already shown encouraging results in hematological malignancies, its application in glioma remains unexplored [78,79,80,81,82,83,84,85,86,87,88,89,90,91,92,93,94,95,96,97,98,99,100,101,102,103,104,105,106,107,108,109,110,111,112,113,114,115,116]. The demonstration of CXCR4 expression in the majority of GBM provides a biological rationale for future therapeutic investigations [77,83,85]. Nevertheless, the marked spatial heterogeneity of receptor expression observed across several studies may represent an important limitation for treatment efficacy and should be considered when selecting candidates for receptor-targeted therapies [78,79,84].

Another interesting point that emerged from the literature is that some studies investigated the role of CXCR4 PET imaging to distinguish glioma from other neoplastic condition. In this setting, Chen et al. demonstrated the utility of [68Ga]Ga-pentixafor PET in differentiating GBM from PCNSL [85]. Although both tumor types exhibited high CXCR4 positivity on immunohistochemistry, lymphoma demonstrated significantly higher tracer uptake and TBR than GBM. Moreover, combining PET-derived quantitative parameters with diffusion MRI substantially improved diagnostic accuracy compared with either modality alone, highlighting the potential role of multiparametric imaging in challenging differential diagnoses [85]. Moreover, Cheng et al. revealed different uptakes between several neoplasms and gliomas with different grade [80].

The relationship between CXCR4-targeted PET uptake and histopathological or molecular features of glioma is complex and does not appear to represent a direct quantitative surrogate of receptor expression. Wang et al. [77] reported higher tracer uptake with stronger CXCR4 immunohistochemical staining, while Lapa et al. [78] found weak CXCR4 expression in PET-negative tumors but no significant quantitative correlation between uptake and immunohistochemical scores in tumors with moderate-to-high expression. Jacobs et al. [79] similarly demonstrated marked intratumoral heterogeneity of CXCR4 staining that was not fully reflected by tracer distribution, whereas Chen et al. [85] reported a significant SUVmax–CXCR4 correlation, stronger in PCNSL than GBM. These findings suggest that CXCR4 PET may better reflect the spatial distribution of CXCR4-related biology than absolute receptor density. Associations with other biomarkers are also inconsistent. Tracer uptake has not been significantly associated with Ki-67, while CXCR4 expression has not consistently correlated with IDH status, MGMT promoter methylation, or overall survival. CXCR4 PET should therefore be considered a complementary biomarker rather than a surrogate of proliferation or established molecular prognosis. Discordance between PET and tissue findings may result from blood–brain barrier (BBB) integrity, perfusion, tracer delivery, vascular activity, partial-volume effects, and spatial mismatch between PET and biopsy samples. Moreover, GBM shows substantial regional heterogeneity in hypoxia, necrosis, vascular proliferation, inflammation, and CXCR4 expression, meaning that limited biopsy samples may not represent the receptor distribution visualized by PET. Overall, higher uptake in HGG and frequent CXCR4 expression in PET-positive regions support the biological rationale for CXCR4 imaging. However, PET uptake should not yet be interpreted as a direct measure of receptor density, proliferation, or prognosis. Larger prospective studies integrating standardized PET quantification, spatially matched histopathology, and comprehensive molecular profiling are needed to define the biological and clinical information most reliably provided by CXCR4-targeted PET.

Although CXCR4 expression is considered a major determinant of tracer uptake, several biological and technical factors may contribute to tracer accumulation and may partly explain the variability observed across studies. In particular, disruption of the BBB may increase tracer delivery to the tumor independently of CXCR4 receptor density, whereas differences in tumor perfusion and vascularity may affect tracer delivery and distribution within the lesion. In addition, inflammatory cell infiltration may influence tracer uptake, as CXCR4 is expressed not only by tumor cells but also by several populations of immune and stromal cells, potentially contributing to the overall signal [117,118,119]. Tracer pharmacokinetics, including blood clearance, tissue distribution, and retention, may further affect measured uptake and may vary according to the specific tracer and imaging protocol [120]. Finally, partial-volume effects, particularly in small lesions, may result in an underestimation of tracer uptake because of limited spatial resolution [121]. Therefore, tracer uptake should not be interpreted as a direct surrogate of CXCR4 expression alone, but rather as the result of the interplay between receptor expression, tumor microenvironment, vascular and BBB characteristics, tracer pharmacokinetics, and imaging-related factors. Consideration of these variables may help reconcile discrepancies between studies and improve the biological interpretation of CXCR4-targeted imaging findings.

The four CXCR4-targeted radiotracers investigated in glioma—[68Ga]Ga-pentixafor, [68Ga]NOTA-NFB, [18F]NOTA-NFB, and [18F]AlF-NOTA-QHY-04—differ in molecular platform, radionuclide, chelation, pharmacokinetics, and receptor-binding properties, limiting direct comparison of their imaging performance. [68Ga]Ga-pentixafor is the most clinically mature tracer. Based on the FC-131/CPCR4 scaffold with a DOTA chelator, it shows high CXCR4 affinity, predominantly renal clearance, and negligible physiological brain uptake. Although absolute glioma uptake is generally modest, the very low cerebral background results in high TBR and excellent lesion conspicuity [78]. Higher uptake has been reported in grade IV than grade III gliomas, and applications include tumor characterization, GBM–PCNSL differentiation, treatment monitoring, and radiotherapy planning. Its related [177Lu]Lu-pentixather platform also provides potential theranostic applications, although these remain investigational [79]. [68Ga]NOTA-NFB, derived from T140 and incorporating NOTA, shows rapid clearance, favorable dosimetry and high tumor-to-background contrast due to minimal physiological brain uptake. However, glioma evidence remains limited to an initial prospective study of eight patients. [18F]NOTA-NFB uses the same molecular platform with [18F], potentially offering greater logistical flexibility [77,86]. [18F]AlF-NOTA-QHY-04 is the newest tracer and combines [18F] with an Al[18F]F-NOTA labelling strategy [80]. Preclinical studies showed high CXCR4 affinity, predominantly renal clearance, and favorable pharmacokinetics. In glioma, uptake was higher in HGG than LGG, but the highest uptake occurred in PCNSL, indicating that CXCR4 uptake is not glioma-specific. Its clinical evidence therefore remains very limited. Overall, [68Ga]Ga-pentixafor has the strongest clinical evidence, whereas the other tracers remain less extensively validated. Their common advantage is negligible physiological brain uptake and consequently high lesion-to-background contrast. However, differences in tracer chemistry, pharmacokinetics, acquisition protocols, and reference regions prevent direct ranking, and none has yet demonstrated a definitive impact on patient management or outcomes. Harmonized head-to-head studies are needed to establish their relative diagnostic and clinical value.

The potential incremental value of CXCR4 PET over amino acid PET should be considered in selected clinical scenarios. While amino acid PET primarily reflects alterations in tumor metabolism and amino acid transport, CXCR4-targeted imaging may provide complementary information on the molecular phenotype and tumor microenvironment through the visualization of CXCR4 expression [78,122,123]. This distinction may be particularly relevant in tumors or lesions in which amino acid uptake is not sufficiently specific to distinguish viable tumor tissue from treatment-related changes, or when conventional metabolic imaging provides equivocal findings. CXCR4 PET may also be of particular interest in the characterization of tumors with heterogeneous or high CXCR4 expression, potentially providing information related to tumor aggressiveness, molecular heterogeneity, and the presence of CXCR4-expressing stromal or inflammatory cells [78,124]. In the post-treatment setting, the combination of metabolic information from amino acid PET with molecular information from CXCR4 PET may therefore offer complementary insights into residual or recurrent disease, although the clinical benefit of such an approach remains to be established. Overall, CXCR4 PET should currently be regarded as a complementary rather than competing imaging modality to amino acid PET, with its greatest potential value likely lying in clinical situations where molecular characterization of the lesion may provide information beyond that obtained from metabolic imaging alone.

Despite these encouraging findings, several limitations currently prevent widespread clinical implementation of CXCR4 PET in glioma imaging. First, the available evidence is characterized by relatively small sample sizes, predominantly single-center experiences, and heterogeneous study designs. Second, multiple radiotracers ([68Ga]Ga-pentixafor, [68Ga]NOTA-NFB, [18F]NOTA-NFB and [18F]AlF-NOTA-QHY-04) with different characteristics have been evaluated, limiting therefore direct comparisons across studies [125,126]. Third, most of the studies considered in the present paper focused on HGG, whereas evidence regarding LGG remains limited. Histopathological validation was not uniformly available and quantitative imaging parameters were not standardized across studies, limiting, therefore, the generalizability of the findings reported in this review. Direct comparisons of TBR values across studies should be interpreted with caution because background reference regions, TBR calculation methods, uptake times, scanner systems, and reconstruction protocols varied or were incompletely reported. Harmonized acquisition and quantification procedures are therefore required before quantitative thresholds can be transferred across centers or tracers. Finally, some publications originated from the same institutions, used comparable imaging protocols, and included overlapping recruitment periods; therefore, partial overlap between patient cohorts cannot be excluded. These studies should not necessarily be interpreted as fully independent datasets. Future studies should therefore focus on prospective multicenter trials employing standardized acquisition protocols and harmonized quantitative analysis. Another important point that needs to be underlined is the fact that direct head-to-head comparisons with amino acid PET tracers are needed to better define the incremental value of CXCR4 imaging for diagnosis, treatment planning, and response assessment. Furthermore, integration of CXCR4 PET with advanced MRI techniques, radiomics, and molecular profiling may improve patient stratification and support precision medicine approaches. Finally, prospective investigations evaluating CXCR4 PET as a companion diagnostic for CXCR4-targeted radionuclide therapy represent one of the most promising future research directions. Another limitation that should be considered is the potential overlap of patient cohorts among studies originating from the same institution or research group. Because CXCR4-targeted imaging remains a relatively specialized technique, several studies may have been conducted at the same centers and during overlapping recruitment periods, raising the possibility that some patients were included in more than one report. Although we attempted to identify overlapping cohorts based on the available information regarding study periods, institutions, sample characteristics, and patient demographics, the possibility of partial or complete overlap cannot be completely excluded, particularly when individual-level data were not available. Such overlap may reduce the statistical independence of the included studies and could lead to an overestimation of the amount and consistency of available evidence if the same patients contribute to multiple analyses. Therefore, the overall findings should be interpreted with caution, and the number of published studies should not necessarily be considered equivalent to the number of independent patient cohorts. Future multicenter studies with clearly defined and non-overlapping populations will be important to provide more robust and generalizable evidence regarding the diagnostic and clinical value of CXCR4-targeted imaging.

Future research should focus on establishing the clinical utility of CXCR4 PET through prospective, adequately powered and preferably multicenter clinical trials. Such studies will be essential to determine whether CXCR4-targeted imaging provides clinically meaningful information beyond established imaging modalities, particularly for diagnosis, treatment response assessment, prognostic stratification, and patient selection for targeted therapies [79,82]. Standardization initiatives will also be important to improve the comparability and reproducibility of CXCR4 PET across centers. Harmonization of acquisition protocols, reconstruction parameters, image analysis, uptake metrics, and reporting criteria could reduce methodological heterogeneity and facilitate the definition of clinically relevant quantitative thresholds [127,128]. In parallel, the integration of CXCR4 PET with radiomics and artificial intelligence may enable the extraction of higher-dimensional imaging features reflecting intratumoral heterogeneity and molecular characteristics that are not readily captured by conventional visual assessment or semiquantitative parameters alone [129,130]. Machine-learning approaches could potentially combine CXCR4 PET features with clinical, molecular, and other imaging biomarkers to improve diagnostic and prognostic models, although such strategies will require external validation and standardized datasets before clinical implementation [128,129]. Finally, the theranostic potential of the CXCR4 axis represents a particularly promising area of investigation. Because CXCR4-targeted imaging may identify tumors with relevant receptor expression, CXCR4 PET could potentially contribute to the selection and monitoring of patients for CXCR4-directed radionuclide or other targeted therapies [79,82,130]. Prospective studies integrating molecular imaging, quantitative analysis, and therapeutic applications will therefore be crucial to determine whether CXCR4 PET can evolve from a promising investigational imaging biomarker into a clinically actionable tool.

## 5. Conclusions

In conclusion, current evidence strongly supports CXCR4-targeted PET imaging as a promising and potentially transformative molecular imaging technique for glioma evaluation, offering distinct advantages over conventional imaging due to its negligible brain uptake and high TBR. Its emerging capabilities in differentiating recurrence, distinguishing histological subtypes, and monitoring treatment response are particularly noteworthy. However, the current evidence is based on small, single-center studies with heterogeneous methodologies. The path to clinical integration requires rigorous validation through large, prospective multicenter trials that standardize acquisition and analysis protocols. These future studies must demonstrate not only diagnostic accuracy but also whether the unique biological insights provided by CXCR4 imaging can improve clinical decision-making and ultimately translate into measurable improvements in patient outcomes, including guiding novel theranostic strategies.

## Figures and Tables

**Table 1:** Main characteristics of the studies considered for the review.

Setting	Glioma Status	N. Glioma Pts.	Study Design	Study Site	Year	First Author [Ref.]
Pretherapy	Primary	8	Prospective	China	2015	Wang Z [77]
Pre- and posttherapy	Primary or recurrent	15	Prospective	Germany	2016	Lapa C [78]
Posttherapy	Recurrent	7	Retrospective	The Netherlands	2022	Jacobs SM [79]
Pretherapy	Primary or recurrent	7	Prospective	China	2024	Cheng K [80]
Pretherapy	Primary	30	Prospective	India	2024	Madan R [81]
Pretherapy	Primary	26	Prospective	Iran	2024	Roustaei H [82]
Pre- and posttherapy	Primary	19	Prospective	India	2024	Waheed A [83]
Pre- and posttherapy	Primary	29	Retrospective	Iran	2025	Dadgar H [84]
Pretherapy	Primary	53	Retrospective	China	2025	Chen Z [85]
Posttherapy	Recurrent	38	Prospective	China	2025	Du Z [86]

N.: number; Pts: patients; Ref.: reference.

**Table 2:** Imaging modalities, radiotracers, administered activities, and main findings of the studies included in the review.

Main Findings	Administered Activity (MBq)	Tracers	Imaging Modality	First Author [Ref.]
[68Ga]NOTA-NFB had a favorable radiation dosimetry profile and was safe for clinical imaging. It had specific glioma uptake with a clean background in normal brain tissue and was more sensitive than [18F]FDG.	1.48–2.96/Kg for [68Ga]NOTA-NFB, 5.55/Kg for [18F]FDG	[68Ga]NOTA-NFB and [18F]FDG	PET/CT	Wang Z [77]
The vast majority of GBM had [68Ga]Ga-pentixafor uptake. TBR for SUVmean and SUVmax were higher for [68Ga]Ga-pentixafor compared to [18F]FET. Histological analysis confirmed CXCR4 expression in areas with high tracer uptake.	83–158 MBq for [68Ga]Ga-pentixafor, 180–229 for [18F]FET	[68Ga]Ga-pentixafor and [18F]FET	PET/CT	Lapa C [78]
Patients showed low to moderate glioma uptake compared with bloodpool activity. A relatively high TBR was demonstrated. Patients showed large intra-tumor variation for CXCR4 staining; however, this variation did not fully correspond with the variation in tracer uptake.	1.5/Kg	[68Ga]Ga-pentixafor	PET/CT	Jacobs SM [79]
Gliomas demonstrated [18F]AlF-NOTA-QHY-04 uptake, without significant difference between LGG and brain metastases of SCLC, both significantly lower than HGG.	4.81/Kg for both	[18F]AlF-NOTA-QHY-04 and [18F]FDG	PET/CT	Cheng K [80]
Radiation dose escalation using [68Ga]Ga-pentixafor PET/CT was feasible and should be further explored.	111–185	[68Ga]Ga-pentixafor	PET/CT	Madan R [81]
The tracer is able to image HGG with excellent visual and semiquantitative diagnostic properties. Mean TBR was significantly higher for WHO grade IV gliomas compared to grade III.	2/Kg	[68Ga]Ga-pentixafor	PET/CT	Roustaei H [82]
[68Ga]Ga-pentixafor uptake was reported in all GBM patients. A significant correlation was found between SUVmean and choline/N-acetil-aspartate ratio. In patients with progression of disease, SUVmax and TBR at follow-up imaging were significantly higher than the corresponding values at baseline and also higher than that noted in the stable patients.	111–148	[68Ga]Ga-pentixafor	PET/CT	Waheed A [83]
Most of gliomas were positive at PET imaging. The median SUVmax of grade IV lesions was significantly higher than grade III lesions. Areas with high uptake showed CXCR4 expression.	114.7–193.5	[68Ga]Ga-pentixafor	PET/CT	Dadgar H [84]
[68Ga]Ga-pentixafor PET in combination with MRI provided valuable diagnostic information in differentiating PCNSL from GBM, especially for atypical PCNSL.	88.1–131.3	[68Ga]Ga-pentixafor	PET/CT	Chen Z [85]
For visual assessment, the accuracy and specificity of [18F]NOTA-NFB were equivalent to [11C]MET and higher than CE-MRI. For quantitative analysis, recurrence revealed higher [18F]NOTA-NFB SUVmax and TBR than treatment-related effects, with higher TBR for recurrence than that of [11C]MET. SUVmax and TBR of both modalities had high AUC for the detection of recurrence.	5.4/Kg for [18F]NOTA-NFB and 9.25/Kg for [11C]MET	[18F]NOTA-NFB and [11C]MET	PET/MRI	Du Z [86]

Ref.: reference; PET: positron emission tomography; CT: computed tomography; Kg: kilogram; MBq: megabecquerel; [18F]FDG: [18F]fluorodeoxyglucose; [18F]FET: [18F]fluoroethyl-l-tyrosine; GBM: glioblastoma; TBR: tumor-to-background ratio; SUV: standardized uptake value; CXCR4: C-X-C chemokine receptor type 4; LGG: low-grade glioma; HGG: high-grade glioma; SCLC: small cell lung cancer; WHO: World Health Organization; MRI: magnetic resonance imaging; PCNSL: primary central nervous system lymphoma; [11C]MET: [11C]methionine; AUC: area under the curve.

**Table 3:** Technical information of the studies included in the review.

Background Reference Region for TBR	Uptake Time	Scanner Model	First Author [Ref.]
Different organs	40 min	Biograph mCT40	Wang Z [77]
The entire contralateral hemisphere at the level of the centrum semiovale	60 min for [68Ga]Ga-pentixafor and 20 min for [18F]FET	Biograph mCT 64	Lapa C [78]
White and gray matter of the contralateral hemisphere	45 min	TruePoint Biograph mCT40	Jacobs SM [79]
Contralateral normal brain tissue	60 min	GEMINI TF Big Bore	Cheng K [80]
/	60 min	ns	Madan R [81]
Contralateral normal brain tissue	60 min	Biograph 6 TrueV	Roustaei H [82]
Cerebellum region in the contralateral side of the tumor site	45 min	GE Discovery 710	Waheed A [83]
Contralateral hemisphere at the level of the centrum semiovale	60 min	Biograph 6 TrueV	Dadgar H [84]
Contralateral grey matter	48.7 ± 10.1 min	Biograph mCT 64	Chen Z [85]
Contralateral hemisphere in an area of normal-appearing brain tissue including white and gray matter	60 min for [18F]NOTA-NFB and 20 min for [11C]MET	Signa PET-MR	Du Z [86]

Ref.: reference; TBR: tumor-to-background ratio; PET: positron emission tomography; min: minutes; [18F]FET: [18F]fluoroethyl-l-tyrosine; [11C]MET: [11C]methionine.

**Table 4:** Summary of the studies included in the review.

Current Evidence Level	Clinical Applications	Weaknesses	Major Strengths	First Author [Ref.]
Prospective pilot/feasibility cohort study	• Primary tumor detection and delineation. • Non-invasive assessment of blood–brain barrier integrity and CXCR4 expression.	• Very small sample size • Preliminary evaluation lacking long-term clinical outcome correlation.	• First prospective human study with [68Ga]NOTA-NFB. • Demonstrated favorable dosimetry, safety, and superior lesion-to-background contrast compared to [18F]FDG.	Wang Z [77]
Prospective diagnostic cohort study	• High-grade glioma visualization. • Delineation of contrast-enhancing tumor regions.	• Small sample size • Heterogeneous patient cohort (primary vs. recurrent tumors). • Lack of quantitative correlation with immunohistochemistry scores.	• Direct comparison with established amino acid tracer [18F]FET. • Histopathological confirmation of CXCR4 expression.	Lapa C [78]
Retrospective case series	• Evaluation of CXCR4 expression as a potential theranostic target ([177Lu]Lu-Pentixather).	• Retrospective design with small sample size • Discrepancy between tracer uptake variation and tissue CXCR4 staining.	• Detailed evaluation of intratumoral CXCR4 spatial heterogeneity. • Assessment of molecular markers and theranostic feasibility.	Jacobs SM [79]
Prospective pilot comparative study	• Glioma grading (differentiation between low-grade and high-grade gliomas). • Differential diagnosis vs. brain metastases.	• Small subset of glioma patients within a broader oncological trial.	• Comparative assessment using a novel CXCR4 antagonist across multiple tumor types.	Cheng K [80]
Prospective Phase II trial without control group	• Biological image-guided radiotherapy planning and dose escalation.	• Did not demonstrate an overall survival benefit despite technical feasibility.	• Prospective Phase II design evaluating therapeutic intervention. • Standardized focus on Grade IV gliomas	Madan R [81]
Prospective diagnostic cohort study	• Diagnostic characterization and grading of high-grade gliomas (WHO Grade III vs. IV).	• Single-center study without direct comparison to amino acid PET.	• Homogeneous focus on high-grade gliomas • Demonstrated high patient-based and lesion-based detection rates.	Roustaei H [82]
Prospective longitudinal cohort study	• Treatment response monitoring following chemo-radiation. • Early detection of disease progression.	• Moderate sample size • Non-uniform changes across PET parameters during follow-up.	• Prospective longitudinal evaluation (pre- and post-radiochemotherapy). • Correlation with MR metrics	Waheed A [83]
Retrospective observational study	• Tumor detection in primary and recurrent high-grade gliomas. • Non-invasive glioma grading.	• Retrospective design. • No significant difference observed in TBR between grade III and IV lesions.	• Assessment of CXCR4 overexpression across both staging and recurrence settings	Dadgar H [84]
Retrospective diagnostic accuracy study with robust internal validation	• Differential diagnosis between glioblastoma and primary CNS lymphoma	• Retrospective design. • Focus restricted to differential diagnosis rather than survival outcomes.	• Largest single cohort for differential diagnosis • Integration of PET parameters with MRI into multiparametric models.	Chen Z [85]
Prospective comparative diagnostic accuracy study	• Differentiating true glioma recurrence from treatment-related effects (pseudoprogression/radiation necrosis).	• Moderate sample size • Prospective study restricted to post-treatment recurrent setting.	• Simultaneous PET/MRI acquisition. • Direct head-to-head comparison with [11C]MET PET and contrast-enhanced MRI.	Du Z [86]

Ref.: reference; PET: positron emission tomography; [11C]MET: [11C]methionine; CXCR4: C-X-C chemokine receptor type 4.

**Table 5:** Comparative overview of CXCR4-targeted PET vs. Amino Acid PET in glioma imaging.

CXCR4-Targeted PET	Amino Acid PET	
Overexpression of CXCR4 chemokine receptors, reflecting tumor aggressiveness, hypoxia, microenvironment crosstalk, and stemness.	Increased amino acid transport via LAT1/LAT2 transporters, independent of blood–brain barrier (BBB) breakdown.	Primary Biological Mechanism
Extremely low to negligible uptake in normal brain parenchyma, offering excellent image contrast.	Very low background uptake in normal brain tissue, providing high target-to-background contrast.	Physiological Brain Background
Emerging molecular modality (primarily evaluated in exploratory/investigational cohorts).	Standard of care in neuro-oncology	Current Clinical Guidelines
Highlights high-grade/hyper-aggressive subregions and areas of vascular disruption.	Delineates non-enhancing infiltrative tumor margins beyond contrast-enhanced MRI.	Tumor Delineation and Biopsy Guidance
Strongly correlates with histological grade; typically shows low uptake in low-grade and robust uptake in high-grade glioma.	Differentiates low-grade from high-grade gliomas, though overlap exists in some IDH-mutant subtypes.	Glioma Grading Capabilities
Promising performance, particularly when integrated with MRI or conventional amino acid PET parameters.	Highly validated and widely used to distinguish treatment-induced changes/radiation necrosis from recurrence.	Pseudoprogression vs. True Recurrence
High potential: Directly guides CXCR4-directed endoradiotherapy (e.g., using [177Lu]Lu-Pentixather).	Limited (primarily diagnostic/monitoring role).	Theranostic Potential
Heterogeneous uptake within tumors; uptake can be influenced by BBB permeability and local inflammation.	Availability of radiotracers, variable uptake in low-grade lesions.	Main Limitations

## Data Availability

Data supporting the reported results can be found using the public scientific databases cited in the text.

## Abbreviations

The following abbreviations are used in this manuscript:

[11C]MET	[11C]methionine
[18F]FDG	[18F]fluorodesoxyglucose
[18F]FET	[18F]fluoroethyl-l-tyrosine
ADC	Apparent diffusion coefficient
CXCL12	C-X-C motif chemokine ligand 12
CXCR4	C-X-C chemokine receptor type 4
GBM	Glioblastoma
HGG	High-grade glioma
IDH	Isocitrate dehydrogenase
LGG	Low-grade glioma
MGMT	O6-Methylguanine-DNA methyltransferase
MRI	Magnetic resonance imaging
MTV	Metabolic tumor volume
OR	Odds ratio
PCNSL	Primary central nervous system lymphoma
PET	Positron emission tomography
SUV	Standardized uptake value
TBR	Tumor-to-background ratio
WHO	World health organization
